# Model containing sarcopenia and visceral adiposity can better predict the prognosis of hepatocellular carcinoma: a multicenter study

**DOI:** 10.1186/s12885-023-11357-5

**Published:** 2023-10-12

**Authors:** Yao Liu, Sirui Fu, Xiangrong Yu, Jinxiong Zhang, Siyu Zhu, Yang Yang, Jianwen Huang, Hanlin Luo, Kai Tang, Youbing Zheng, Yujie Zhao, Xiaoqiong Chen, Meixiao Zhan, Xiaofeng He, Qiyang Li, Chongyang Duan, Yuan Chen, Ligong Lu

**Affiliations:** 1 Zhuhai Interventional Medical Centre, Zhuhai People’s Hospital (Zhuhai Hospital Affiliated with Jinan University), No. 79 Kangning Road, Zhuhai, 519000 Guangdong Province China; 2 Department of Radiology, Zhuhai People’s Hospital, Zhuhai Hospital Affiliated with Jinan University, Zhuhai, 519000 Guangdong Province China; 3 Department of Biostatistics, School of Public Health, Southern Medical University, No. 1023-1063 Shatai South Road, Guangzhou, 510515 Guangdong Province China; 4 Guangdong Provincial Key Laboratory of Tumor Interventional Diagnosis and Treatment, Zhuhai Institute of Translational Medicine, Zhuhai People’s Hospital (Zhuhai Hospital Affiliated with Jinan University), Zhuhai, 519000 Guangdong China; 5 Interventional Diagnosis and Treatment Department, Nanfang Hospital, Southern Medical University, Guangzhou, China; 6 Department of Radiology, Shenzhen People’s Hospital, Shenzhen, China; 7 Department of Interventional Treatment, Zhongshan City People’s Hospital, No. 2, Sunwen East Road, Zhongshan, 528400 Guangdong Province China

**Keywords:** Hepatocellular carcinoma, Sarcopenia, Visceral adiposity, Muscle steatosis

## Abstract

**Aim:**

This study aimed to explore whether the addition of sarcopenia and visceral adiposity could improve the accuracy of model predicting progression-free survival (PFS) in hepatocellular carcinoma (HCC).

**Methods:**

In total, 394 patients with HCC from five hospitals were divided into the training and external validation datasets. Patients were initially treated by liver resection or transarterial chemoembolization. We evaluated adipose and skeletal muscle using preoperative computed tomography imaging and then constructed three predictive models, including metabolic (Model^MA^), clinical–imaging (Model^CI^), and combined (Model^MA−CI^) models. Their discrimination, calibration, and decision curves were compared, to identify the best model. Nomogram and subgroup analysis was performed for the best model.

**Results:**

Model^MA−CI^ containing sarcopenia and visceral adiposity had good discrimination and calibrations (integrate area under the curve for PFS was 0.708 in the training dataset and 0.706 in the validation dataset). Model^MA−CI^ had better accuracy than Model^CI^ and Model^MA^. The performance of Model^MA−CI^ was not affected by treatments or disease stages. The high-risk subgroup (scored > 198) had a significantly shorter PFS (*p* < 0.001) and poorer OS (*p* < 0.001).

**Conclusions:**

The addition of sarcopenia and visceral adiposity improved accuracy in predicting PFS in HCC, which may provide additional insights in prognosis for HCC in subsequent studies.

**Supplementary Information:**

The online version contains supplementary material available at 10.1186/s12885-023-11357-5.

## Introduction

Hepatocellular carcinoma (HCC) was the sixth most frequently diagnosed cancer and the third leading cause of cancer-related deaths worldwide in 2020 [1]. Resection and transarterial chemoembolization (TACE) are two of the first-line treatments for patients with HCC without extrahepatic metastasis or macrovascular invasion [2, 3]. There are some prognostic factors for patients with HCC, such as liver function and clinical stage [4]. In recent years, indicators related to systemic metabolism have attracted more and more attention. Some studies have indicated that sarcopenia or visceral adiposity is associated with decreased survival in patients with HCC [5–8]. Based on previous studies, we know that sarcopenia and visceral adiposity were independent risk factors for poor prognosis of HCC. However, extremely few studies have used both sarcopenia and visceral adiposity simultaneously in the construction of HCC prognostic model. To sum up the above, our team believes that it is necessary and meaningful to incorporate sarcopenia and visceral adiposity into the predictive model construction of prognosis for HCC. The simultaneous inclusion of them may help us more thoroughly understand the systemic metabolism of patients with HCC. In addition, some articles have also tried to build predictive models, but lacking reliable verification may lead to overfitting in previous research [9–11].

Therefore, in this multicenter study, we aimed to explore whether the addition of sarcopenia and visceral adiposity improved accuracy in predicting progression-free survival (PFS) in HCC. By this process, we hope to provide additional insights in prognosis for HCC.

## Material and methods

### Patient selection

**Fig. 1:**
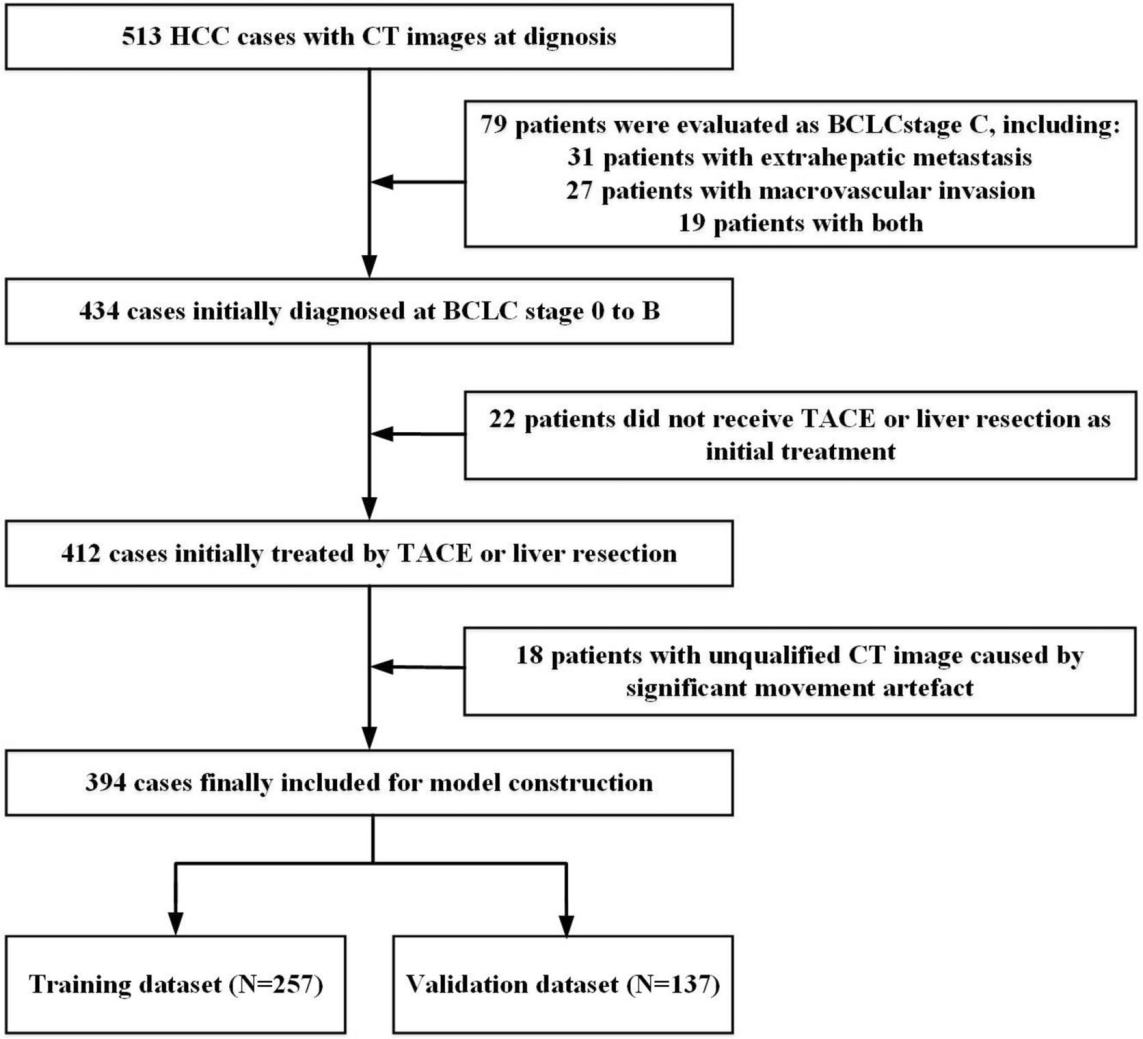
The inclusion and exclusion flowchart showing patient selection for this study. We screened 513 patients from five hospitals. After the inclusion and exclusion criteria were evaluated, 394 patients were divided into the training (*n* = 257) and external validation (*n* = 137) datasets

We recruited patients from five hospitals in China. Patients initially diagnosed with HCC between July 2006 and November 2016 were included and followed up until December 2018. HCC was diagnosed clinically or pathologically according to existing guidelines. The inclusion criteria were as follows: (1) patients with CT examination recorded at diagnosis, (2) patients initially treated by liver resection or TACE according to the recommended guidelines, (3) patients undergoing liver resection with negative pathological results of margins, and (4) patients who developed progressive disease (PD) during treatment or were regularly followed up without PD at least one year unless death occurred. The exclusion criteria were as follows: (1) patients who were classified as Barcelona Clinic Liver Cancer (BCLC) stage C at the time of diagnosis., (2) patients received other initial treatments, such as ablation or percutaneous ethanol injection and (3) patients with significant movement artifacts on CT images. Therefore, we enrolled 394 patients initially treated with TACE or liver resection for HCC in our study (Fig. 1).

The study protocols were approved by the Ethics Committee of Zhuhai People’s Hospital. The requirement for informed consent to use the patients’ data for medical research was waived since the data was collected retrospectively. All patient records and information were anonymized and de-identified prior to analysis.

### Treatments and follow-up

The initial treatment option, including liver resection or TACE, was determined by a multidisciplinary team based on the recommended guidelines, patients' liver function, and treatment intention [12, 13]. For liver resection, negative pathological results of margins was required. For TACE, super-selective embolization with lipiodol and chemotherapy drug was operated under the guidance of digital subtraction angiography. Follow-up visits occurred every 4–6 weeks in the first year until the patient died or the end of the study, including chest radiography, abdominal CT/magnetic resonance imaging (MRI), and necessary laboratory tests. When residual viable tumors or new lesions were found, the patient were treated according to their individual situation and guidelines after multidisciplinary discussion. Patients without PD were censored by the end of this study, which is December 2018.

### Outcomes

To evaluate tumor response to treatment, CT and MRI scans were analyzed based on the modified Response Evaluation Criteria in Solid Tumors (mRECIST criteria) for HCC [14]. The primary endpoint was progression-free survival (PFS), defined as the time from the initial treatment to PD. The secondary endpoint was overall survival (OS), which was calculated as the time from the initial treatment to death.

### Clinical factors and radiological characteristics

Candidate factors are listed in Table 1. Additionally, we considered the following factors: (1) neutrophil-to-lymphocyte ratio (NLR) [15]; (2) HCC spatial location, including the lobe (classified as left, right, or cross-sectional) and surface (whether lesions adjacent to the liver capsule were present); and (3) nine radiological signs extracted from the preoperative CT images: fusion lesions, invasive shape, capsule integrity, capsule breakthrough, corona enhancement, corona with low attenuation, mosaic architecture, nodule-in-nodule architecture, and enhancement ratio of HCC. The radiological signs were assessed by two independent radiologists with more than 10 years of clinical work experience form the central hospital. If disagreement occurred, a third radiologist with more than 20 years of clinical work experience performed re-evaluation. Finally, conclusions were made by discussion of the three radiologists. The intraclass correlation coefficients for radiological signs ranged from 0.885 to 0.987.

**Table 1:** Baseline demographics of patients included in the study

	**Total (*N* = 394)**	**Training dataset (*N* = 257)**	**Validation dataset (*N* = 137)**	***p*-value**
**Metabolic factors:**				
**SAT(cm**^**2**^**)**	101.26(43.70–158.82)	102.60(43.15–162.06)	98.76(44.82–152.70)	0.5296
**SATI (cm**^**2**^**/m**^**2**^**)**	32.84(8.69–56.99)	33.66(9.12–58.02)	31.31(7.90–54.72)	0.3597
**VAT(cm**^**2**^**)**	92.29(23.97–160.61)	95.20(24.74–155.66)	86.82(22.83–150.81)	0.2464
**VATI (cm**^**2**^**/m**^**2**^**)**	36.48(14.94–58.02)	36.78(14.74–58.82)	35.90(15.28–56.52)	0.7015
**VSR**	0.98(0.05–1.91)	1.09(0.06–2.12)	0.89(0.40–1.38)	0.1569
**MAT(cm**^**2**^**)**	132.78(106.27–159.31)	132.20(105.01–159.39)	133.90(108.63–159.17)	0.5363
**MATI (cm**^**2**^**/m**^**2**^**)**	47.05(38.79–55.31)	46.63(38.28–54.98)	47.84(39.80–55.88)	0.1668
**IMAC**	-0.61(-0.84- -0.38)	-0.62(-0.86- -0.38)	-0.59(-0.81- -0.37)	0.1888
**Clinical factors**				
**Age (year)**	55.43 (43.10–67.76)	56.30 (44.02–68.66)	53.90 (41.53–66.32)	0.064
**Height (m)**	1.68 (1.61–1.75)	1.68 (1.61–1.75)	1.67 (1.60–1.74)	0.219
**NLR**	2.85 (0.63–5.07)	2.79 (0.49–5.09)	3.00 (0.94–5.10)	0.443
**TBIL (umol/L)**	18.16 (5.98–30.34)	18.00 (4.57–31.43)	18.39 (8.94–27.84)	0.790
**Alb (g/L)**	40.30 (34.60–46.00)	39.92 (33.97–45.87)	41.04 (35.89–46.19)	0.063
**ALT (U/L)**	48.63 (3.99–93.27)	49.62 (0.44–98.8)	46.77 (12.1–81.44)	0.546
**Sex (N)**				0.459
Male	326 (82.74%)	210 (81.71%)	116 (84.67%)	
female	68 (17.26%)	47 (18.29%)	21 (15.33%)	
**Child–Pugh score (N)**				0.916
5	251 (63.70%)	163 (63.42%)	88 (64.23%)	
6	90 (22.84%)	58 (22.57%)	32 (23.36%)	
7	40 (10.15%)	28 (10.89%)	12 (8.76%)	
8	13 (3.29%)	8 (3.11%)	5 (3.65%)	
**AFP level (ng/mL)**				0.560
< 20	166 (42.13%)	113 (43.97%)	53 (38.69%)	
20–400	105 (26.65%)	65 (25.29%)	40 (29.20%)	
> 400	123 (31.22%)	79 (30.74%)	44 (32.12%)	
**HBV (N)**				0.129
Negative	17 (4.32%)	14 (5.45%)	3 (2.19%)	
Positive	377 (95.68%)	243 (94.55%)	134 (97.81%)	
**BCLC stage (N)**				0.775
0	46 (11.68%)	28 (10.89%)	18 (13.14%)	
A	243 (61.68%)	161 (62.65%)	82 (59.85%)	
B	105 (26.64%)	68 (26.46%)	37 (27.01%)	
**Treatment(N)**				0.863
Liver resection	140(35.53%)	85(33.07%)	55(40.15%)	
TACE	254(64.47%)	172(66.93%)	82(59.85%)	
**Imaging factors:**				
**Lesions number(N)**				0.223
1	268(68.02%)	176(68.48%)	92(67.15%)	
2	60(15.23%)	35(13.62%)	25(18.25%)	
≥ 3	66(16.75%)	46(17.90%)	20(14.60%)	
**Max-diameter(mm)**	62.36(22.70–102.02)	64.35(23.80–104.90)	58.65(20.85–96.45)	0.218
**Fusion lesion(N)**				0.175
No	249(63.20%)	173(67.32%)	76(55.47%)	
Yes	145(36.80%)	84(32.68%)	61(44.53%)	
**HCC capsule(N)**				0.113
Absent	69(17.51%)	36(14.01%)	33(24.09%)	
Non-intact	231(58.63%)	160(62.26%)	71(51.82%)	
Intact	94(23.86%)	61(23.73%)	33(24.09%)	
**halo sign(N)**				0.476
No	322(81.73%)	219(85.21%)	103(75.18%)	
Yes	72(18.27%)	38(14.79%)	34(24.82%)	
**Mosaic (N)**				0.338
No	105(26.65%)	74(28.79%)	31(22.63%)	
Yes	289(73.35%)	183(71.21%)	106(77.37%)	
**Cirrhosis(N)**				0.999
No	176(44.67%)	115(44.75%)	61(44.53%)	
Yes	218(55.33%)	142(55.25%)	76(55.47%)	

### Sarcopenia and visceral adiposity

We determined the area of skeletal muscle and abdominal adipose tissue from the cross-sectional CT images at the third lumbar vertebra (L3), using Slice-O-Matic 4.3 software (Tomovision, Montreal, QC, Canada) [16]. Based on previous reports, the skeletal muscle area (psoas major, rectus abdominis, and quadratus lumborum) was identified with thresholds of –29 to 150 Hounsfield units (HUs). Abdominal adipose tissue was identified using the following thresholds: –190 to –30 HU for subcutaneous adipose tissue and –150 to –50 HU for visceral adipose tissue [16]. We measured the original area indicators for subcutaneous adipose tissue and visceral adipose tissue as subcutaneous adipose tissue (SAT) and visceral adipose tissue (VAT). The visceral fat deposition was evaluated by visceral-to-subcutaneous adipose tissue area ratio (VSR), which was calculated as follows: VSR = VAT/SAT [7]. In addition, intramuscular adipose tissue content (IMAC) was used to examine the muscle quality at the L3 level using the following formula: IMAC = CT attenuation value of the multifidus muscles (HU)/CT attenuation value of the subcutaneous fat (HU) [17]. A higher IMAC indicated that more adipose tissue was deposited in the skeletal muscle (muscle steatosis) (Additional Fig 1) [18].

For skeletal indictors, we used the skeletal muscle index (SMI) which was standardized by height in meters squared as reported [16]. For adipose indicators, considering the controversy in standardization, we tested whether they should be standardized by height in meters squared(cm^2^/m^2^) [19, 20]. By this process, we extracted standardized index indicators: subcutaneous adipose tissue index (SATI) and visceral adipose tissue index (VATI). The we tested whether the original or standardized indicators were better for predicting PFS by the Akaike information criterion (AIC) [21].

### Statistical analyses

Eligible patients from three hospitals were used as the training dataset, and those from the remaining two hospitals were included as the external validation dataset. For the comparison of these two datasets, continuous variables are expressed as means (standard deviation) or medians (25th and 75th percentiles) when appropriate and were compared using Student’s t-test or Wilcoxon rank-sum test. All categorical and ordinal variables are displayed as counts (percentages) and were compared using the Wilcoxon rank-sum test, Pearson’s χ^2^ test, or Fisher’s exact test.

To evaluate the value of metabolic variables in the prediction of prognosis, we constructed three models using stepwise Cox regression sequentially. Differences among the models were compared using the likelihood ratio test. The predictive accuracy of these models was assessed by both the discrimination measured by the receiver operating characteristic (ROC) curve and the calibration evaluated by the calibration plot. The clinical utility was also evaluated using decision curve analysis (DCA). For ease of use, a nomogram was constructed for the selected model. Subgroup analysis according to treatment and Barcelona Clinic Liver Cancer (BCLC) stage was performed to further evaluate the performance of the selected best model. After classifying the patients using the median risk score of the selected best model, we compared the PFS and OS between the low-, moderate-, and high-risk groups using Kaplan–Meier plots and log-rank tests.

All tests were two-sided, and a *p*-value < 0.05 was considered statistically significant. Statistical analyses were performed using R statistical package (version 4.1.2, Vienna, Austria, http://www.r-project.org/).

## Results

### Patients’ baseline characteristics

This retrospective study included 394 patients with HCC diagnosed firstly from July 2006 and November 2016. Three hospitals’ patients were used as the training dataset (*N* = 257) and remaining two hospitals’ patients were used as the external validation dataset (*N* = 137). In total, 177 and 78 patients developed PD in the training and validation datasets, respectively. The patients initially treated by liver resection and TACE were 140 (35.5%) and 254 (64.5%). During follow-up, 255 patients (training dataset: 177; validation dataset: 78) showed PD, 122 patients (training dataset: 88; validation dataset: 34) died. For BCLC stages, 46 (training dataset: 28; validation dataset: 18), 243 (training dataset: 161; validation dataset: 82), and 105 (training dataset: 68; validation dataset: 37) patients had BCLC 0, A, and B stages, respectively. Clinical indicators, there were no statistical differences in the baseline characteristics between patients in the training and validation datasets (Table 1).

### Model development and comparison

Among the investigated clinical and imaging factors, Cox regression analysis revealed that total bilirubin, BCLC stages, number of lesions, and HCC capsule were significantly associated with PFS (Supplementary Tables 1 and 2). The AIC of area indicators was 1680, which was less than 1683 for index indicators, so we chose area indicator for the model construction with clinical and imaging indicators. We constructed three models to explore the correlation between metabolic disorders and the prognosis of HCC. The metabolic model (Model^MA^) incorporates metabolic disorder factors, such as SAT, VAT, VSR, SMI and IMAC. The clinical–imaging model (Model^CI^) contained clinical and imaging factors, such as treatment method, BCLC stage, total bilirubin (TBIL), capsule integrity and number of lesions. The constituent indicators of the combined model (Model^MA−CI^) were as follows: treatments, SAT, VAT, VSR, SMI, IMAC, TBIL, number of lesions, capsule integrity, and BCLC stages. The formulas for each model were shown in the Supplementary material.

**Fig. 2:**
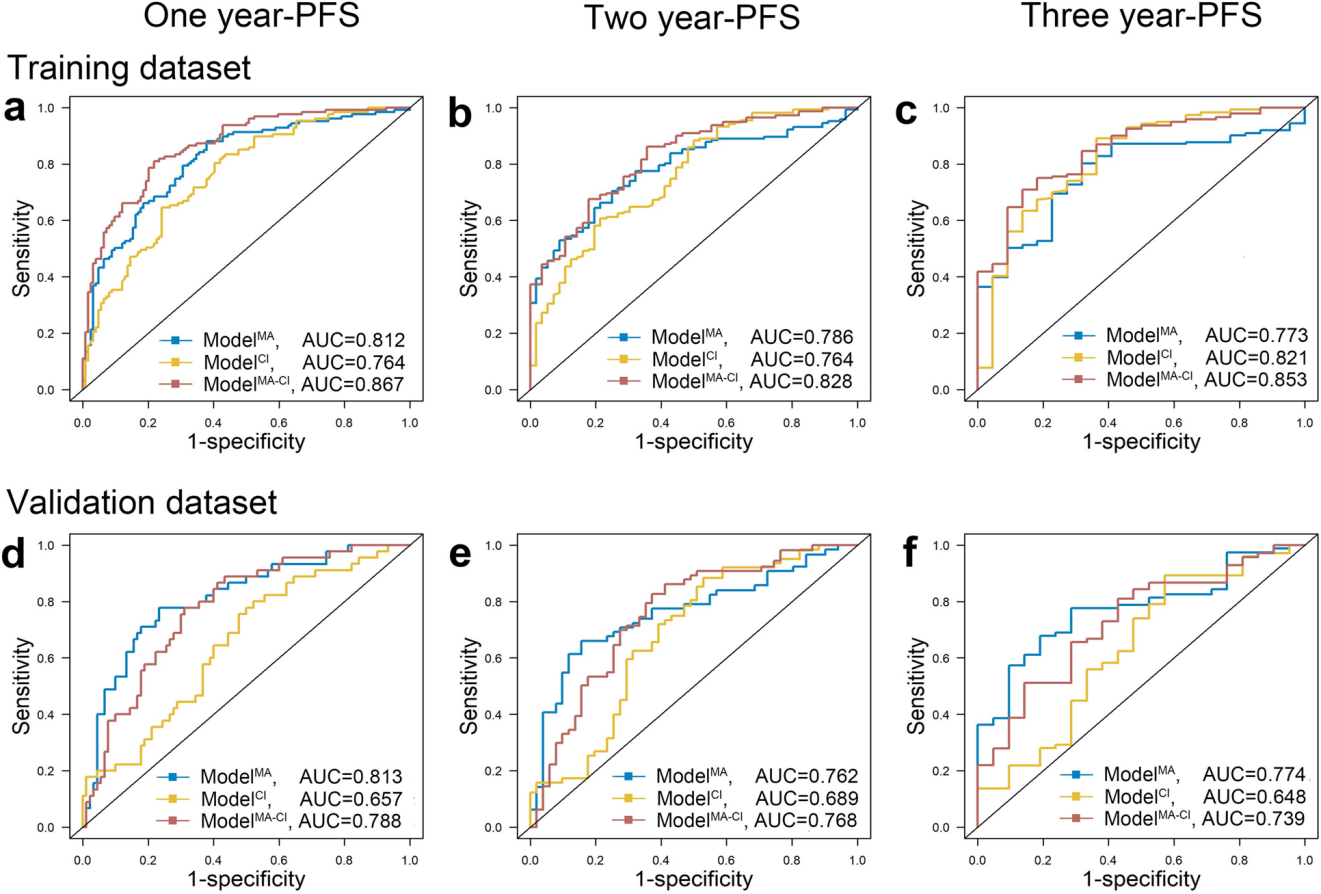
Time-dependent ROC curve: Model comparisons with the areas under the curve (AUCs). **a-c** The AUCs of the metabolic, clinical–imaging, and combined models in the training datasets. **d-f** The AUCs of the metabolic, clinical–imaging, and combined models in the validation datasets

**Fig. 3:**
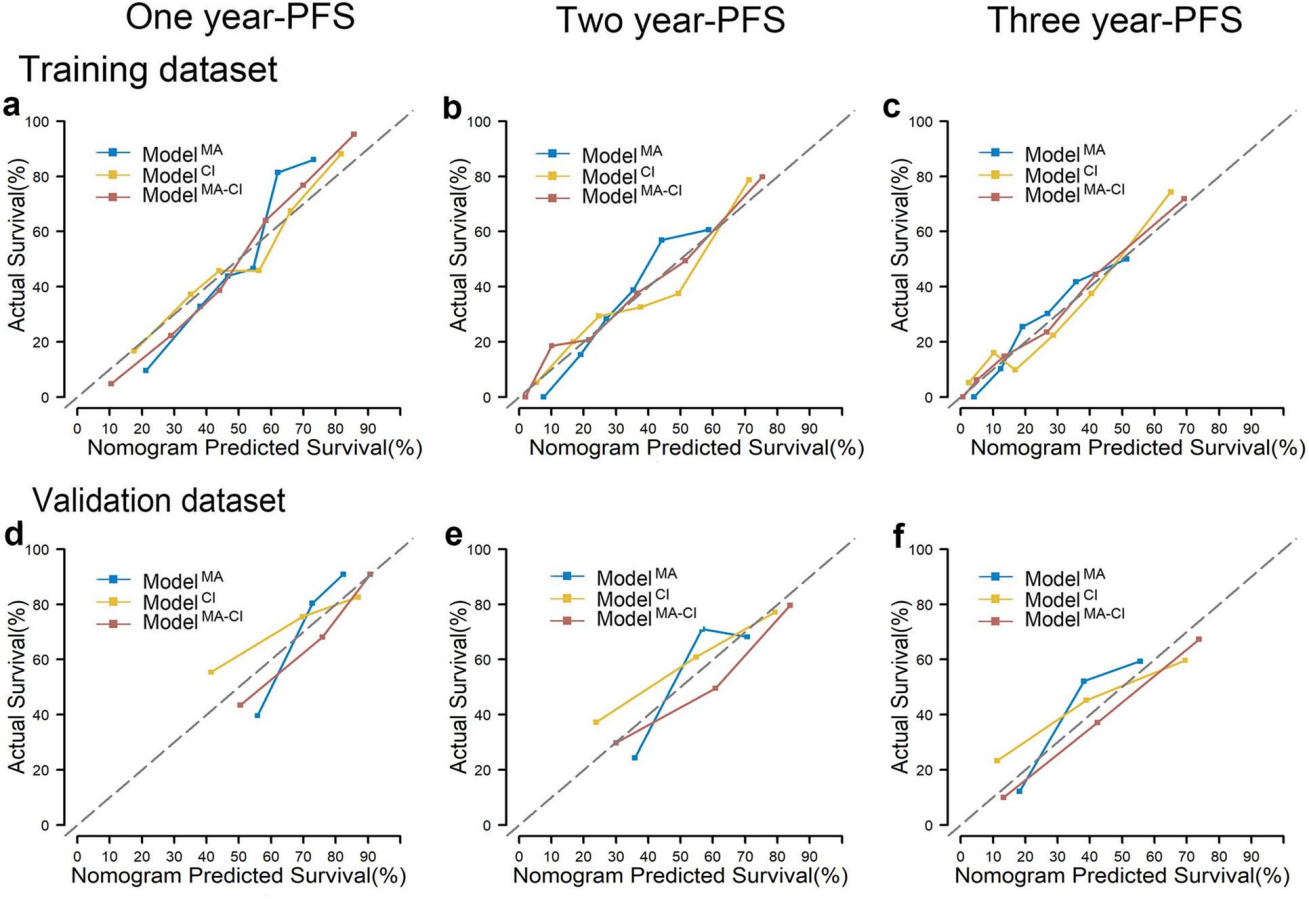
Model comparisons with the calibrations. **a-c** The calibrations are displayed for the training datasets in one, two, and three years. **d-f** The calibrations are displayed for the validation datasets in one, two, and three years

**Fig. 4:**
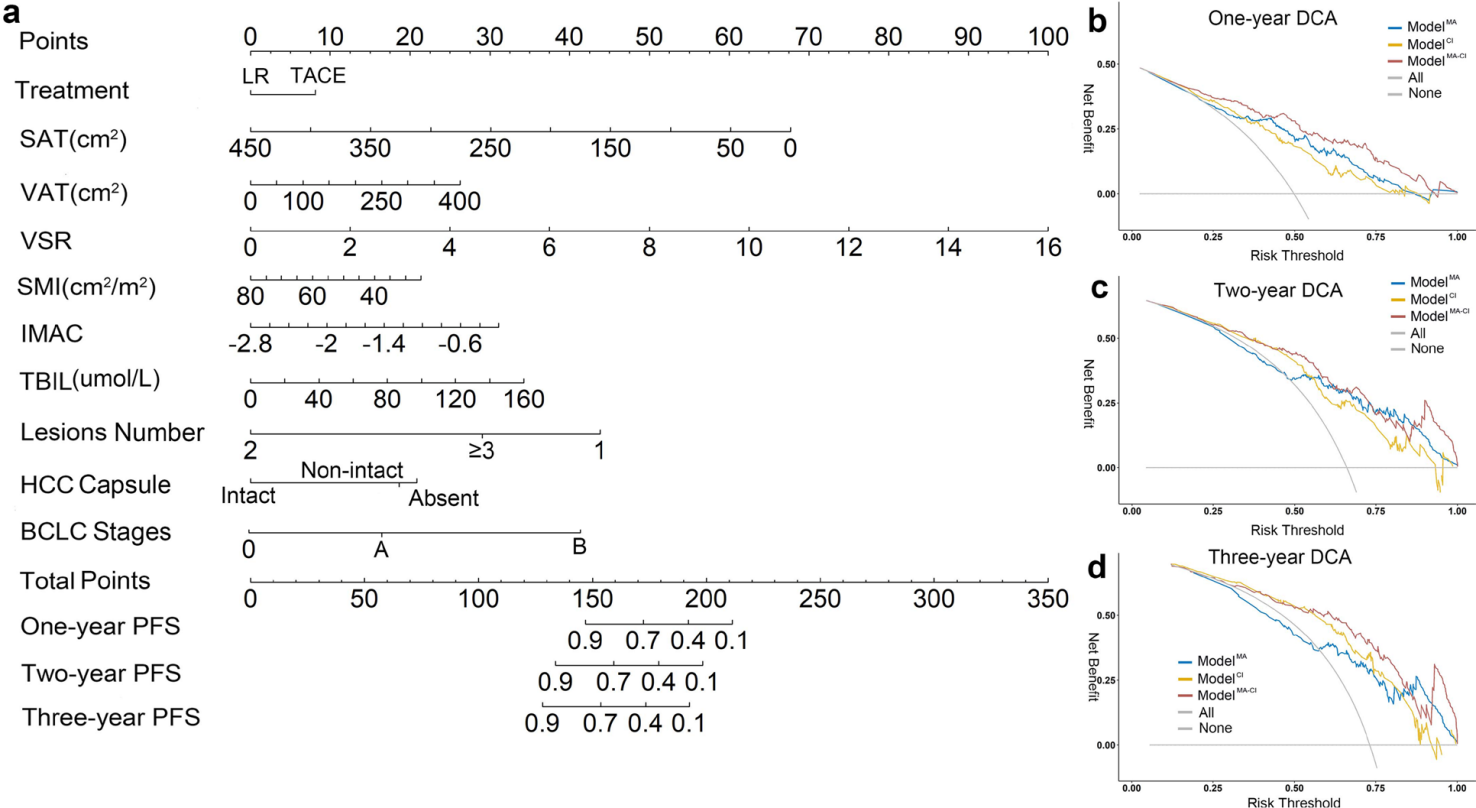
Nomogram and decision curve of Model^MA−CI^. **a** The nomogram of Model^MA−CI^. **b-d** The decision curve for Model^MA^, ModelCI and Model^MA−CI^ in one, two, and three years

We compared the models’ discrimination and calibration to identify the best model. Regarding discrimination, the areas under the curve (AUC) for one-, two-, and three-year PFS in the combined model was better than those in the clinical–imaging model and the metabolic model in the training dataset (0.812, 0.786, and 0.773; 0.764, 0.764, and 0.821; and 0.867, 0.828, and 0.853, respectively). Similar results were observed in the validation dataset (0.813, 0.762, and 0.774; 0.657, 0.689, and 0.648; and 0.788, 0.768, and 0.739, respectively). The AUC of the combined model was better than the clinical–imaging model (Fig. 2). The integrate-AUC and C-index for three models were shown in Supplementary Fig. 3. Regarding calibration, the performance of the combined model was better than that of the metabolic model and similar to that of the clinical–imaging model (Fig. 3). Based on these results, the combined model was identified as the optimal model, and we constructed a nomogram for it. The DCA cures for the three models are shown in Fig. 4.

### Subgroup analyses

Subgroup analysis according to sex (Male and Female), BCLC stages (0, A, and B stage) and treatments (TACE or resection) showed that the selected combined model had similar performance across different subgroups. For different sex, Male vs Female, the AUCs of one, two, and three years of PFS were 0.826 vs 0.844 (*p* = 0.732), 0.807 vs 0.717 (*p* = 0.234) and 0.825 vs 0.611 (*p* = 0.065). For different treatments, liver resection vs TACE, the AUCs of one, two, and three years of PFS were 0.844 vs 0.817 (*p* = 0.544), 0.776 vs 0.774 (*p* = 0.978) and 0.768 vs 0.767 (*p* = 0.985). For different BCLC stages, stage 0 + A vs stage B, the AUCs of one, two, and three years of PFS were 0.815 vs 0.843 (*p* = 0.554), 0.787 vs 0.779 (*p* = 0.920) and 0.788 vs 0.787 (*p* = 0.981). (Supplementary Fig. 2).

### Survival analysis

**Fig. 5:**
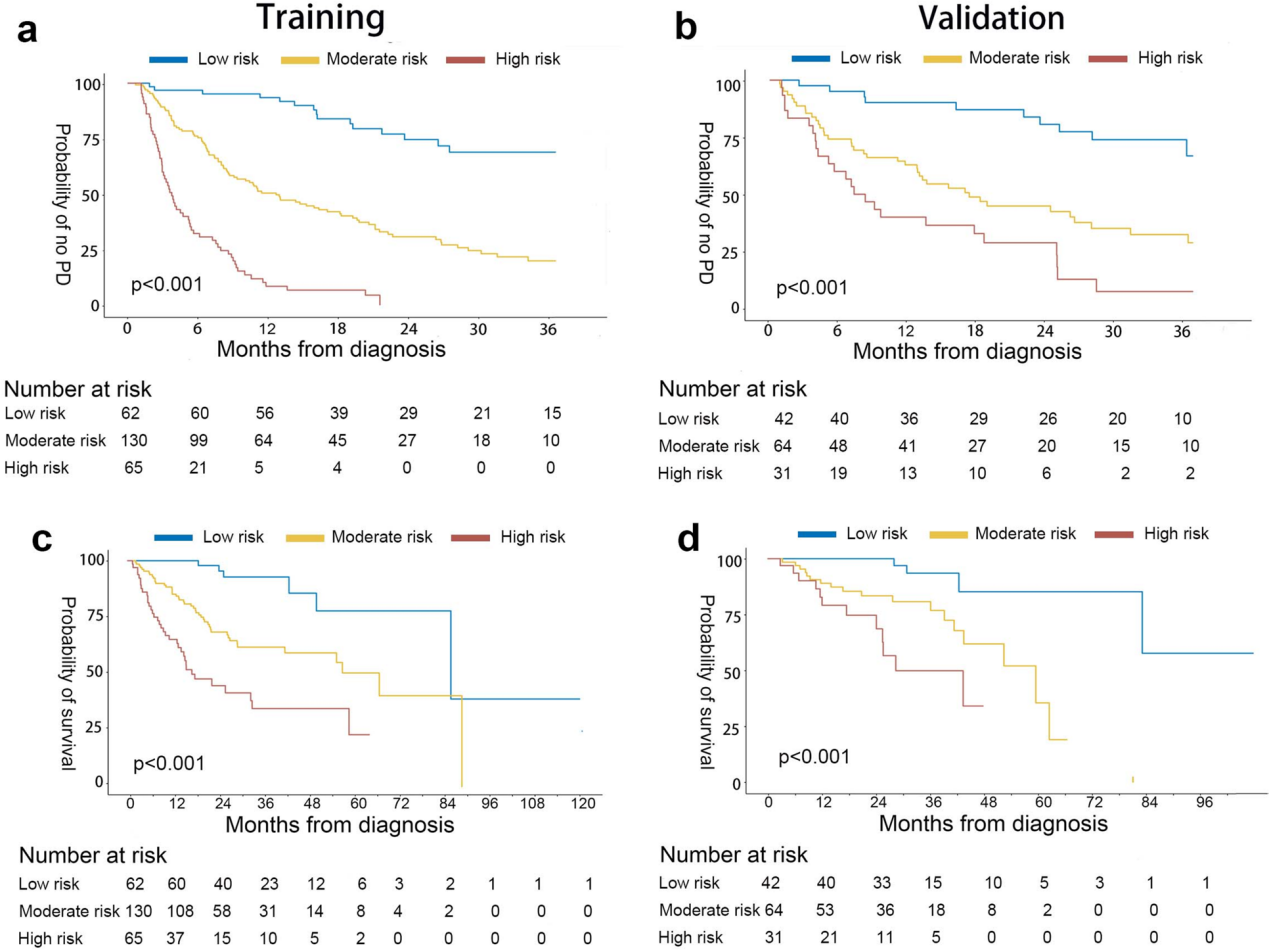
Kaplan–Meier curves for survival analysis. **a** Progression-free survival in the training dataset. **b** Progression-free survival in the validation dataset. **c** Overall survival in the training dataset. **d** Overall survival in the validation dataset

By using the 25th and 75th percentiles (were 172 and 198 respectively) of combined model (Model^MA−CI^), we divided the patients into low-, moderate-, and high-risk groups, and the result showed the low-risk group had significantly longer PFS and more favorable OS. Both the moderate and high-risk groups were compared with the low-risk group, respectively. For PFS in training dataset: HR = 4.629 (95%CI: 2.676–8.000) in moderate-risk group, and HR = 16.002 (95%CI: 8.888–28.810) in high-risk group, *p* < 0.001 (Fig. 5a). For PFS in external validation dataset: HR = 3.682 (95%CI: 1.833–7.392) in moderate-risk group, and HR = 6.584 (95%CI: 3.134–13.829) in high-risk group, *p* < 0.001; median: Infinite vs. 554 vs. 247 days (Fig. 5b). For OS, the results were similar, in the training dataset: HR = 4.507 (95%CI: 1.922–10.565) in moderate-risk group, and HR = 10.761 (95%CI: 4.496–25.755) in high-risk group, *p* < 0.001 (Fig. 5c). in external validation dataset: HR = 5.822 (95%CI: 1.694–20.008) in moderate-risk group, and and HR = 12.813 (95%CI: 3.456–47.504) in high-risk group, *p* < 0.001; median: Infinite vs. 1740 vs. 832 days (Fig. 5d).

## Discussion

In this multicenter study, we constructed a combined model (Model^MA−CI^) to predict prognosis of HCC. In this model, the addition of sarcopenia and visceral adiposity improved the performance for both discrimination and calibration.

In clinical, preoperative prognostic evaluation is mainly based on patients' clinical factors, such as tumor stage [22] and potential liver function. When it comes to imaging indicators, we always focused on the exploration of tumor lesions and peritumoral zones but paid relatively little attention to sarcopenia and visceral adiposity which may reflect patients' nutritional status. In previous research, obesity has been shown to be a risk factor for various cancers, mainly in the digestive system, especially pancreatic[23] and liver cancers [24]. Simultaneously, sarcopenia and obesity increase the mortality rate of cirrhosis [25]. Similar results were observed in patients with liver cancer who underwent liver transplantation [26] or liver resection [27].

In this study, the AUC of the combined model was better than the clinical–imaging model in the external validation dataset. At the same time, the calibration of the combined model was also better than the metabolic model in the external validation dataset. Based on the two points of appeal, the combined model which incorporates clinical, imaging indicators, sarcopenia and visceral adiposity has a comprehensive and promising capacity in predicting prognosis. Meanwhile, the performance of the combined model was not influenced by different treatments or disease stages, which further proved its robustness under different conditions.

In our study, comparing the combined model and the clinical–imaging model using AUCs, we found that the addition of metabolic indicators improved the discrimination of the model. The related metabolic indicators are explained as follows: visceral adipose and subcutaneous adipose tissues are the two main types of adipose tissue. Inadequate subcutaneous fat is an independent risk factor for poor cancer prognosis in studies of relevant oncological microenvironment [28]. Subcutaneous adipocytes may play a beneficial role in metabolism, which is similar to the results of our study [29]. Adipose tissue is considered a secretory organ that produces pro-inflammatory and anti-inflammatory cytokines and adipokines. A high VSR value indicates that the fat distribution tends to be observed in the visceral area, which is often related to a poor prognosis [30]. By analyzing CT images of the patient before treatment, it was possible to determine the condition of the tumor zone and evaluate the patient’s nutritional metabolism. From our study, the condition of muscle and adipose tissue is correlated with the prognosis of HCC, so provided nutritional support may be beneficial to the prognosis [31]. Although it is not clear whether preoperative and postoperative interventions, such as nutritional therapy and rehabilitation, can improve postoperative results by changing obesity or muscle reduction, they are still worthy of attention.

In addition, comparing the combined model and the metabolic model by calibration showed that the addition of clinical–imaging indicators improved calibration. A higher BCLC stage, higher TBIL level [32], and more tumor nodules are associated with a poorer prognosis for HCC. A high TBIL level often indicates liver dysfunction. The capsule of liver cancer is often formed by the compression of the surrounding normal liver tissue. Intact capsules are often present in tumors with a low degree of malignancy, indicating that the tumor and other tissues are well demarcated and less aggressive [33].

Our study has some limitations. First, due to regional reason, almost all the patients included in study had a history of hepatitis B. Whether our results were suitable for HCC related to hepatitis C still needed to be tested. Second, BCLC stages C patients were not included in our study, because according to the guidelines them cannot undergo TACE or liver resection. Third, our study investigated the prognosis of initial treatment after diagnosis. In our original data, patients with other treatments (like ablation therapy) were limited, so they were not included in our study to control bias. However, in the follow-up study, we will expand the source of cases and further explore. Fourth, for variable of the number of nodules in the nomogram, the risk of the “single lesion” is higher than the “two lesions”, it may because the data of our study may have a bias. In future, we will improve this limitation in subsequent studies. Finally, in our study, skeletal muscle and adipose tissue were evaluated by two-dimensional imaging. Stereoscopic three-dimensional measurements will certainly provide more prognostic information.

## Conclusion

In our study, we established a combined model based on sarcopenia and visceral adiposity by using multi-center data. Our results showed that the addition of them improved accuracy in predicting PFS in HCC. This finding may provide new insights into the prognosis of HCC in subsequent studies.

### Supplementary Information

**Additional file 1. **

## Data Availability

Due to the privacy of patients, the data related to patients cannot be available for public access but can be obtained from the corresponding author on reasonable request approved by the institutional review board of Zhuhai People’s Hospital (llg0902@sina.com).

## Abbreviations

AIC: Akaike information criterion
AUC: Area Under the curve
BCLC: Barcelona clinic liver cancer
CT: Computed tomography
DCA: Decision curve analysis
HCC: Hepatocellular carcinoma
HUs: Hounsfield units
IMAC: Intramuscular adipose tissue content
SATI: Subcutaneous adipose tissue index
SMI: Skeletal muscle index
VATI: Visceral adipose tissue index
TACE: Transarterial chemoembolization
MRI: Magnetic resonance imaging
OS: Overall survival
PD: Progressive disease
PFS: Progression-free survival
TBIL: Total bilirubin
