# Supplementary material for: Model containing sarcopenia and visceral adiposity can better predict the prognosis of hepatocellular carcinoma: a multicenter study
Source: BMC Cancer. 2023 Oct 12;23:969. doi: 10.1186/s12885-023-11357-5 (PMC10568831; doi:10.1186/s12885-023-11357-5)
Supplement: Supplementary file 1 — Additional file 1. [file 12885_2023_11357_MOESM1_ESM.docx]

**Model containing sarcopenia and visceral adiposity can better predict the prognosis of hepatocellular carcinoma: A multicenter study**

**Additional Materials**

**Table of Contents**

Number of Tables: 2

Number of Figures: 3

**Formula of models:**

Model^MA^：

h(t,x)/h(t,0)=exp(0.372×*I*(Group=2)+(-0.008)×SAT+0.003×VAT+0.321×VSR+(-0.013)×SMI+0.602×IMAC)

Model^CI^：

h(t,x)/h(t,0)=exp(0.411×*I*(Group=2)＋0.008×TBIL+(-2.215)×*I*(Lesions number group=2)+(-0.822)×*I*(Lesions number group=3)+(-0.205)×*I*(HCC Capsule=1)+(-0.986)×*I*(HCC Capsule=2)+0.953×*I*(BCLC Stages=1)+2.400×*I*(BCLC Stages=2))

Model^MA-CI^：

h(t,x)/h(t,0)=exp(0.3900×*I*(Group=2)+(-0.007)×SAT+0.003×VAT+0.299×VSR+(-0.019)×SMI+0.573×IMAC＋0.010×TBIL+(-2.100)×*I*(Lesions number group=2)+(-0.707)×*I*(Lesions number group=3)+(-0.106)×*I*(HCC Capsule=1)+(-0.997)×*I*(HCC Capsule=2)+0.797×*I*(BCLC Stages=1)+1.987×*I*(BCLC Stages=2))

**Description of variables**

Group: Treatment (1=liver resection; 2= TACE)

TBIL: Total bilirubin

HCC Capsule(0=intact; 1=non-intact; 2=absent)

BCLC Stages(0=0 stage; 1=A stage; 2=B stage)

SAT: Subcutaneous adipose tissue

VAT: Visceral adipose tissue

VSR=VAT/SAT

SMI: Skeletal muscle index

IMAC: Intramuscular adipose tissue content


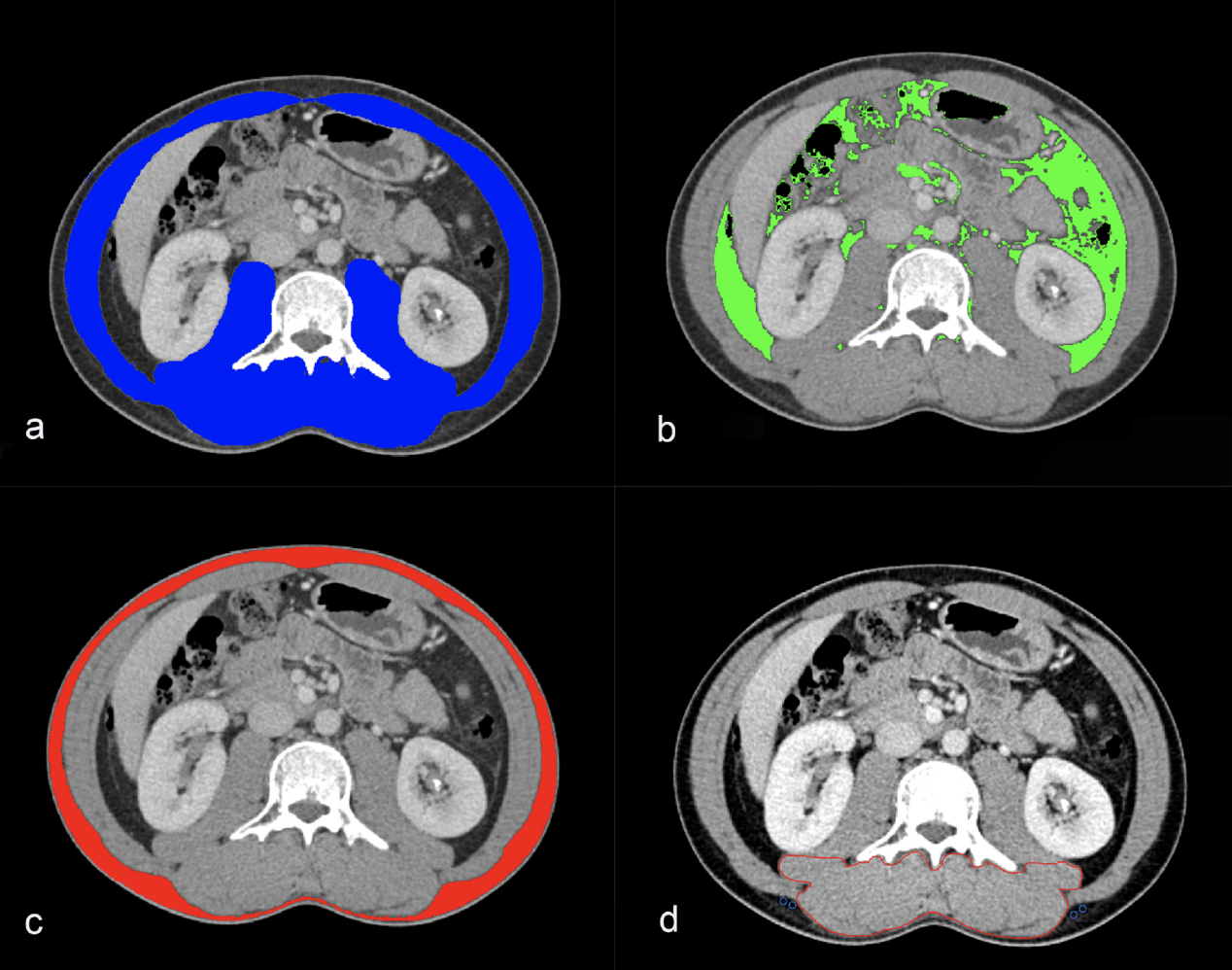


**Supplementary Figure 1 Metabolic indicators were determined by the preoperative CT images at L3 level.**

**a** Skeletal muscle area was quantified using a CT attenuation value of –29 to 150 HUs. **b** Visceral adipose tissue area was quantified using attenuation values of –150 to –50 HU. **c** Subcutaneous adipose tissue area was identified and quantified using CT attenuation values of –190 to –30 HU. **d** CT attenuation values of subfascial muscular tissue in the multifidus muscle and subcutaneous fat (four small circles) are examined to calculate the intramuscular adipose tissue content. CT, computed tomography; HU, Hounsfield unit.

**
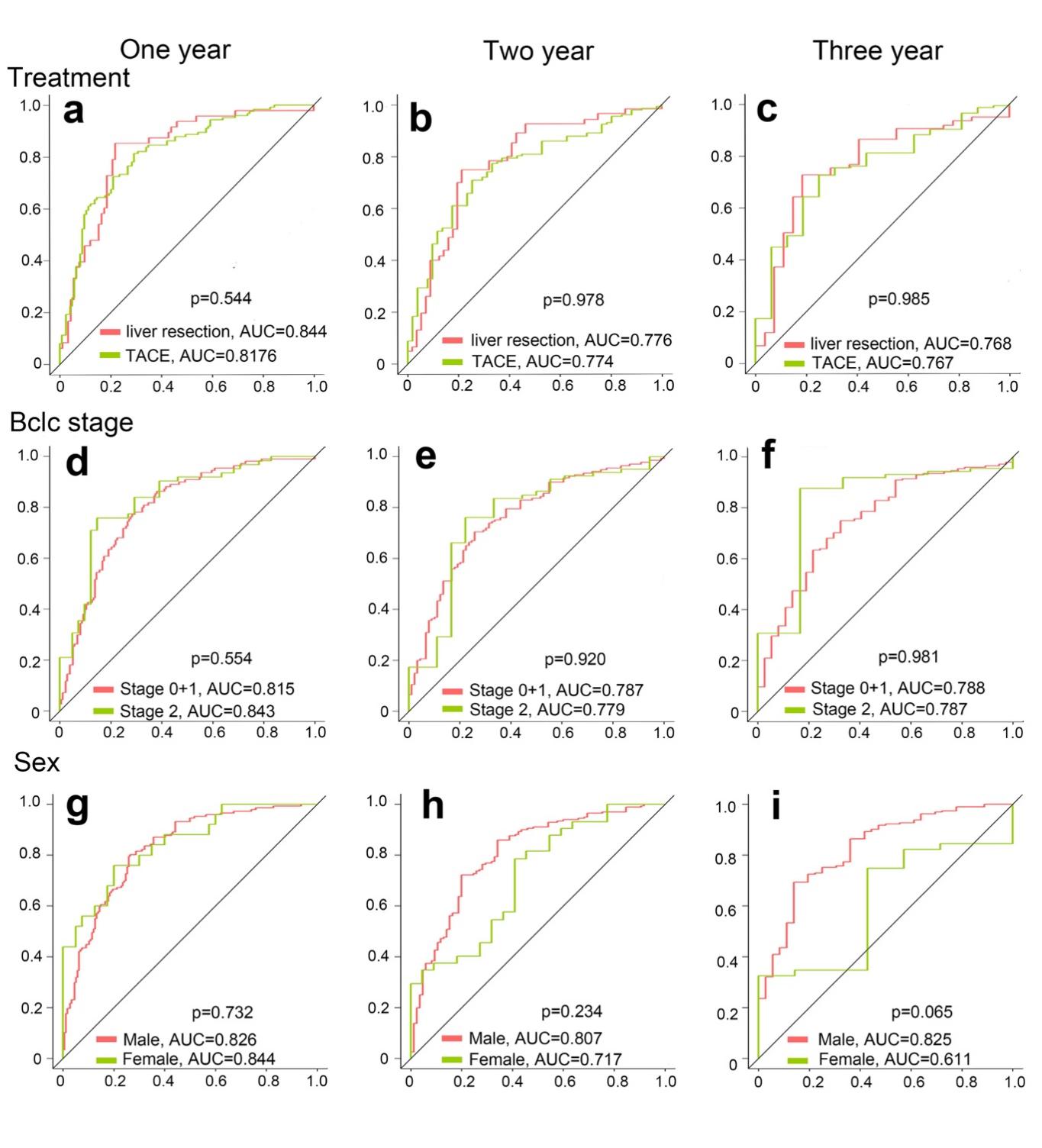
**

**Supplementary Figure 2 Subgroup analysis for different BCLC staging and treatments.**

1. **c** For different treatments, TACE or resection, the AUCs were not statistically difference at one, two, and three years.

**d-f** For different BCLC stages,0, A, and B stage, the AUCs were not statistically difference at one, two, and three years.

**g-i** For different SEX, Male or Female, the AUCs were not statistically difference at one, two, and three years.

| **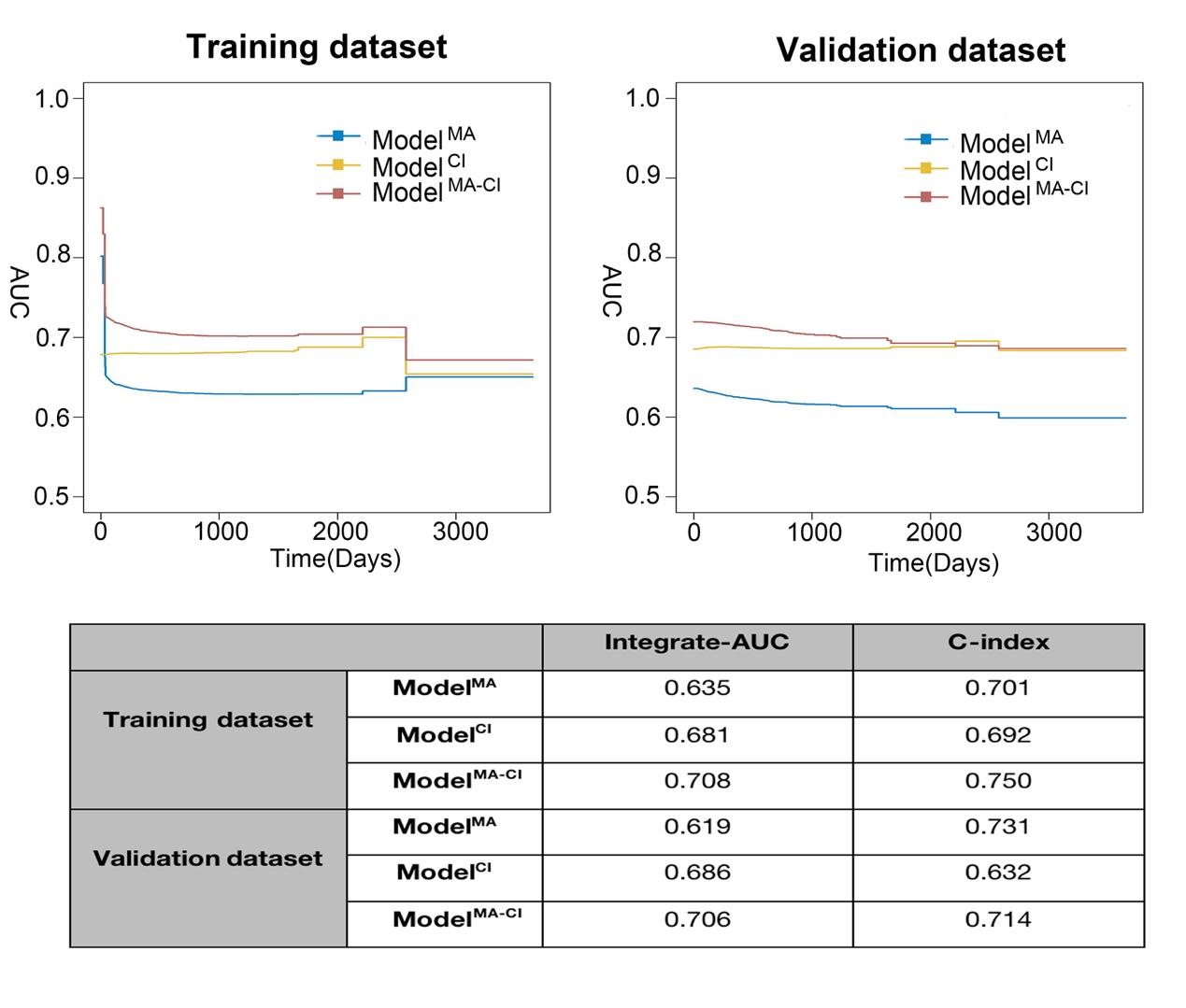**  **Supplementary Figure 3 The integrated AUC and c-index for PFS.**  **Supplementary Table 1. Univariate cox regression analyses for PFS of the training dataset** | | |
| --- | --- | --- |
| **Factors** | **HR (95% CI)** | ***p*-value** |
| **Clinical factors** |  |  |
| Age**(year)** | 1.003 (0.990–1.015) | 0.669 |
| Sex**(N)** | 0.988 (0.676–1.443) | 0.949 |
| Height**(m)** | 0.547(0.074 –4.053) | 0.555 |
| Weight**(kg)** | 1.978(0.581–8.682) | 0.268 |
| BCLC stage |  |  |
| 0 |  | Reference |
| A | 1.666 (0.953–2.913) | 0.073 |
| B | 2.607(1.436–4.732) | 0.002 |
| Child-Pugh class | 1.435 (0.956–2.156) | 0.082 |
| Treatments | 1.582 (1.142–2.189) | 0.006 |
| HBV | 0.909 (0.464–1.780) | 0.780 |
| TBIL**(U/L)** | 1.005(0.997–1.013) | 0.021 |
| ALT**(U/L)** | 1.003(1.001–1.006) | 0.251 |
| AFP (ng/mL) |  |  |
| <400 |  | Reference |
| ≥400 | 1.315 (0.956–1.810) | 0.093 |
| Neutrophil-lymphocyte ratio | 1.007 (0.952–1.065) | 0.813 |
| **Imaging factors** |  |  |
| Lesions number |  |  |
| 1 |  | Reference |
| 2 | 0.446 (0.255–0.779) | 0.004 |
| ≥3 | 2.364 (1.648–3.391) | <0.001 |
| Maximum diameter | 1.008 (1.005–1.012) | <0.001 |
| Location | 2.097 (1.458–3.017) | <0.001 |
| Adjacent to liver surface | 1.303 (0.785–2.161) | 0.305 |
| Fusion lesion | 2.158 (1.585–2.938) | <0.001 |
| HCC capsule |  |  |
| Absent |  | Reference |
| Non-intact | 0.862 (0.579–1.285) | 0.467 |
| Intact | 0.329 (0.193–0.560) | <0.001 |
| HCC capsule breakthrough | 2.159 (1.438–3.242) | <0.001 |
| Mosaic architecture | 2.146 (1.496–3.078) | <0.001 |
| low density halo sign | 1.076 (0.703–1.645） | 0.737 |
| Cirrhosis | 1.149 (0.851-1.550) | 0.364 |
| **Metabolic** **factors** |  |  |
| SAT(cm^2^) | 0.995(0.992–0.998) | <0.001 |
| VAT(cm^2^) | 1.003(1.000–1.006) | 0.047 |
| VSR | 1.593(1.393–1.821) | <0.001 |
| SMI (cm^2^/m^2^) | 0.983(0.964–1.001) | 0.069 |
| IMAC | 1.089(0.590–2.010) | 0.786 |
| PFS: progression-free survival; AFP: alpha-fetoprotein; BCLC: Barcelona Clinic Liver Cancer; CI: confidence interval; HBV: hepatis B virus; HCC: hepatocellular carcinoma; OR: odds ratio; TBIL: total bilirubin; ALT: alanine transaminase; SAT:subcutaneous adipose tissue; VAT:visceral adipose tissue; VSR=VAT/SAT; SMI:skeletal muscle index; IMAC:intramuscular adipose tissue content | | |

| **Supplementary Table 2. Multivariate cox regression analyses for PFS of the training dataset** | | |
| --- | --- | --- |
| **Factors** | **HR (95% CI)** | ***p*-value** |
| **Clinical factors** |  |  |
| TBIL | 1.010 (1.001–1.019) | 0.031 |
| BCLC stage |  |  |
| 0 |  | Reference |
| A | 2.218 (1.156–4.254) | 0.017 |
| B | 7.295(2.110–25.218) | 0.002 |
| Treatment | 1.476(1.055–2.063) | 0.023 |
| **Imaging factors** |  |  |
| Lesions number |  |  |
| 1 |  | Reference |
| 2 | 0.123 (0.041–0.368) | <0.001* |
| ≥3 | 0.493 (0.174–1.403) | 0.185 |
| HCC capsule |  |  |
| Absent |  | Reference |
| Non-intact | 0.899 (0.578–1.399) | 0.639 |
| Intact | 0.369 (0.206–0.662) | 0.001 |
| **Metabolic** **factors** |  |  |
| SAT | 0.993(0.988–0.997) | 0.002 |
| VAT | 1.003(1.000–1.006) | 0.047 |
| VSR | 1.348(1.154–1.575) | <0.001 |
| SMI (cm^2^/m^2^) | 0.981(0.961–1.002） | 0.081 |
| IMAC | 1.773(0.741–4.241) | 0.198 |
| PFS: progression-free survival; CI: confidence interval; HCC: hepatocellular carcinoma; OR: odds ratio; TBIL: total bilirubin; SAT:subcutaneous adipose tissue; VAT:visceral adipose tissue; VSR=VAT/SAT; SMI:skeletal muscle index; IMAC:intramuscular adipose tissue content | | |
